# Antibody responses to tick-borne encephalitis virus non-structural protein 1 and whole virus antigen–a new tool in the assessment of suspected vaccine failure patients

**DOI:** 10.1080/20008686.2019.1696132

**Published:** 2019-12-03

**Authors:** Bo Albinsson, Bengt Rönnberg, Sirkka Vene, Åke Lundkvist

**Affiliations:** a Department of Medical Biochemistry and Microbiology, Zoonosis Science Centre, Uppsala University, Uppsala, Sweden; b Laboratory of Clinical Microbiology, Uppsala University Hospital, Uppsala, Sweden; c Department of Microbiology, The Public Health Agency of Sweden, Solna, Sweden

**Keywords:** Tick-borne encephalitis (TBE), vaccine failure, vaccine breakthrough, vaccine-preventable diseases, vaccines and immunization, antibody detection, serology, diagnostics

## Abstract

We report a new tool for improved serological diagnostics in suspected tick-borne encephalitis (TBE) vaccine failure cases. Due to an increase in the incidence of disease as well as the number of vaccinees, specific and simplified diagnostic methods are needed. Antibody responses to TBE-virus (TBEV) non-structural protein 1 (NS1) are detectable post TBEV infection but not post vaccination. We have used samples from 14 previously confirmed Swedish TBEV vaccine failure patients to study antibody responses against NS1 and whole virus antigens, respectively. Our conclusion is that the detection of antibodies directed to TBEV NS1 antigen is a useful tool to considerably simplify and improve the quality in investigations regarding suspected TBEV infection in vaccinated patients.

## Introduction

Long-term neurological sequelae and case fatalities can occur in tick-borne encephalitis (TBE) patients [1,2], but also mild cases are probably common and often remain undiagnosed. The disease typically follows a biphasic pattern with flu-like symptoms in the first phase and a second phase with symptoms ranging from meningitis to encephalitis. Transmission to humans occurs almost exclusively from tick bites, although viral transmission via milk products has been shown.

In the second phase of the disease, when neurological symptoms are present, laboratory diagnosis is highly dependent on the detection of TBE-virus (TBEV)-specific IgM and IgG in blood and/or cerebrospinal fluid (CSF) [3]. Viral RNA can normally be found in patient samples only during the early first phase of the disease. Immune-compromised patients with delayed antibody responses may have a prolonged viremic phase that, in rare cases, enables TBEV-RNA detection.

**Figure 1.**
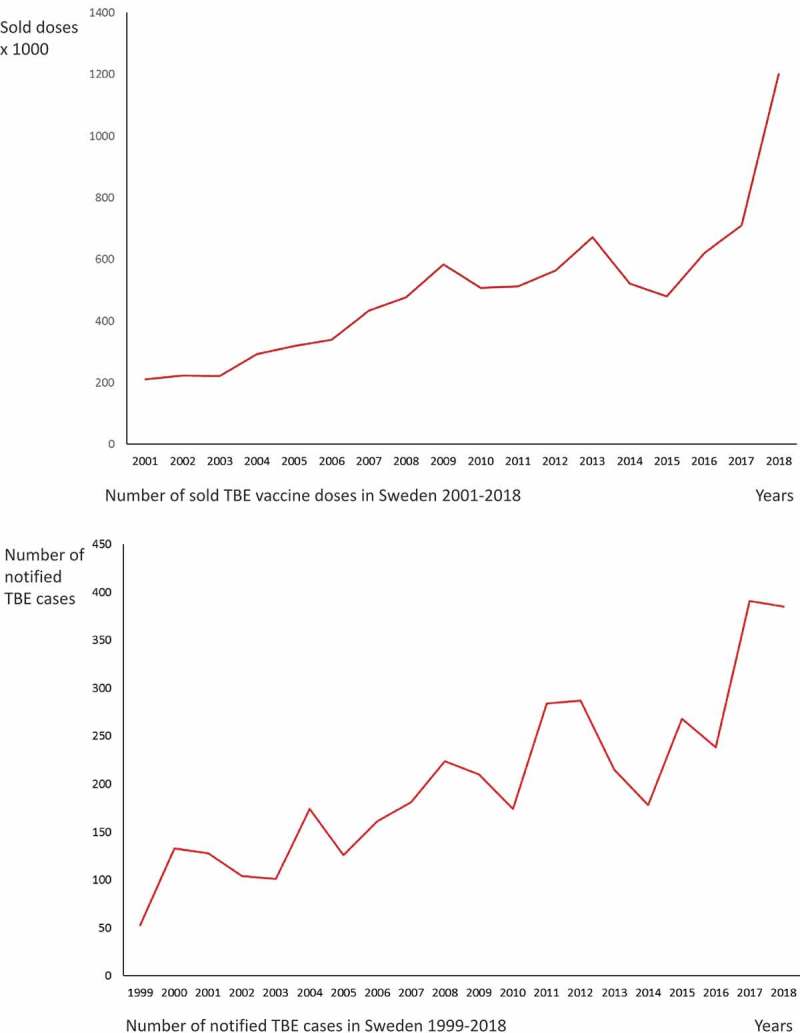
(a) Number of sold TBE vaccine doses in Sweden 2001-2018. (b) Number of notified TBE cases in Sweden 1999-2018. Source: The Public Health Agency of Sweden.

TBE is an important and growing public health problem in Europe; France reported a marked increase in TBE cases in 2016, and in Finland, the number of TBE cases has not only more than doubled during the last decade, but the virus has also spread to new geographical areas. The Netherlands, previously TBE-free, most recently reported its emergence. In Sweden, the number of notified cases is increasing and reached a record-high in 2017 (391 cases), with almost the same level in 2018 (385 cases) (Figure 1) [4]. Effective vaccines are available, but vaccine failures occur [5,6]. The number of sold vaccine doses has also increased during the same period and reached 1.2 million doses in 2018, which is double the amount sold the years before (Figure 1). The commercial or in-house serological tests that are commonly used are not designed to separate antibody responses induced by infection from those induced by vaccination, and interpretation of serological patterns is most challenging. This is even more the case if the patient has been vaccinated in close proximity to the onset of suspected TBE illness during the TBE season. As TBE vaccination is becoming more common, this diagnostic problem will increase even further. A diagnostic tool that can distinguish antibody responses induced by TBEV infection from those induced by vaccination is thus highly desirable.

Our recently published method for detection of non-structural protein 1 (NS1) and whole virus (WV) antibodies to TBEV using TBEV suspension multiplex immunoassay (SMIA) was proven to efficiently differentiate between antibodies induced by infection and vaccination. All but two (48/50) samples from TBEV-infected patients had antibodies to NS-1 antigen as compared to only three serum samples from the vaccinated group (3/50). In one case, it is possibly due to a TBEV infection during the study period [7]. Vaccination alone does not give rise to NS1 antibodies, as NS1 is not present in the vaccine preparation. Thus, antibody responses (IgM or IgG) to NS1 are due to a current or a previous TBEV infection.

Our aim in this study was to determine whether the presence of antibodies directed to the TBEV NS1 antigen could prove to be a useful tool for the diagnosis of TBEV infection in vaccinated patients. Samples from 14 previously confirmed TBE vaccine failure patients were tested in order to study the antibody responses against NS1 and WV antigens, respectively.

## Materials and methods

### TBEV SMIA

The Luminex-based TBEV SMIA for the detection of antibodies to TBEV WV and NS1 antigens in serum was reported in 2018 [7]. In this study, also CSF samples were included and analysed at a dilution of 1/20.

### Samples and patients

Seventeen serum and 18 CSF samples drawn between 2006 and 2011 from 14 patients and previously analyzed at the Public Health Agency of Sweden were tested. The samples were primarily analyzed at different clinical microbiology laboratories in Sweden before they were sent to the Public Health Agency for further investigation. The mean age of the patients was 56 years (6–68) and the majority were men (male/female ratio 80%/20%). The time from disease onset to the first sample was 4–30 days (mean: 11.5). In three cases, this information was not available, but these samples were all drawn from symptomatic patients, i.e. during the acute phase of the disease. All patients had a documented history of previous TBEV vaccination according to the schedule. Due to clinical and laboratory-suspected TBEV infection despite a completed vaccination schedule, the samples were sent to the Public Health Agency. After serological testing including IgG and IgM detection in sera and CSF together with neutralization test, they were all considered to be TBE vaccine failure cases (Table 1).

**Table 1.** Summary of results for each vaccination failure patient.

Patient No.	Age at disease (years)	Sex	Number of vaccine doses	Time from last vaccine dose to disease	No of sampling points^a^	Neutralization titer	IgM and IgG antibodies to WV antigen in sera	IgM antibodies to NS1 antigen in sera or CSF	IgM antibodies to NS1 antigen in sera alone
**1**	63	Male	4	2 years	2	10, >160	Positive	Positive	Negative
**2**	67	Male	3	2 years	1	640	Positive	Positive	Positive
**3**	39	Male	2	3 months	1	80	Positive	Positive	Positive
**4**	21	Male	2	5 months	1	40–80	Positive	Positive	Positive
**5**	6	Male	3	1 year	1	>160	Positive	Positive	Positive
**6**	67	Female	3	Unknown	2	640, 1280	Positive	Positive	Positive
**7**	55	Female	2	4 months	1	160	Positive	Positive	Positive
**8**	52	Female	5	3 years	1	5	Positive	Negative	Negative
**9**	68	Male	4	5 months	1	80	Positive	Positive	Positive
**10**	64	Male	3	1 year	2	20, 80	Positive	Positive	Positive
**11**	74	Male	4	3 years	1	160	Positive	Positive	Positive
**12**	62	Male	4	3 years	1	n.d.	Positive	Negative	Negative
**13**	72	Male	3	1 year	1	320	Positive	Positive	Positive
**14**	76	Male	3	2 years	1	80–160	Positive	Negative	Negative
							14/14	11/14	10/14

^a^Three patients had more than one sample. Only one of the sampling points has been scored for each patient. See the Results section for more detailed explanation.

### Vaccine failure definition

Vaccine failure was defined as a TBEV infection despite adherence to the recommended vaccination schedule with at least two doses [8].

## Results

Eleven out of 14 patients (78%) were found IgM antibody positive to TBEV NS1 antigen in serum or CSF by the TBEV SMIA, while all 14 patients (100%) tested positive for IgM and IgG to TBEV WV antigen (Table 1). Patients 1, 6 and 10 had two sampling points. Blood and CSF samples were available from both sampling points. The time span was from 12 days to 1 month counted from the first acute sample. Patient 1 was NS1 IgM positive only in the second CSF sample taken 12 days after the first acute sample. Patient 6 was NS1 IgM positive in both serum samples (negative CSF). Patient 10 was NS1 IgM positive both in serum samples and in the second CSF sample. Each patient was scored only once.

## Discussion

All 14 patients were found IgM and IgG positive against TBEV WV antigen, supporting exposure to TBEV from infection or vaccination. Eleven out of the 14 patients tested IgM positive to TBEV NS1 antigen in serum or in CSF, strongly supporting a current or recent TBEV infection. Ten patients were found IgM positive to TBEV NS1 antigen in serum alone.

Three of the patients had no detectable antibodies to NS1 antigen either in serum or in CSF at the time of sampling. They all had IgM and IgG responses to WV antigen, suggesting that the patients were immune competent enough to respond to TBEV vaccination. If these patients developed anti-NS1 antibodies later or not at all is not known since there is no information on any follow-up tests or clinical data available. For that reason, any possible correlation between lack of anti-NS1 antibody response and more serious illness could not be investigated.

In Sweden, there is an unexpected discrepancy between increased vaccination coverage and the number of diagnosed cases. The reason for this is not known but could be caused by a number of reasons including increased patient and doctor awareness, changed climate factors and/or host–vector relationships.

We believe that the TBEV SMIA method is very useful as a new and relatively simple tool in the diagnostics of suspected vaccination failure cases. We will now continue using the method, both for larger study materials and for clinical samples in the laboratory, internal as well as external samples. The TBEV SMIA method will also be used in a study for seroprevalence of TBEV NS1 and WV antibodies in blood donors in different parts of Sweden, with an attempt to investigate the proportion of TBE-vaccinated individuals compared with TBEV exposed. Very limited data on this matter exist today.
